# Ghrelin Improves Functional Survival of Engrafted Adipose-Derived Mesenchymal Stem Cells in Ischemic Heart through PI3K/Akt Signaling Pathway

**DOI:** 10.1155/2015/858349

**Published:** 2015-03-24

**Authors:** Dong Han, Wei Huang, Sai Ma, Jiangwei Chen, Lina Gao, Tong Liu, Rongqing Zhang, Xiujuan Li, Congye Li, Miaomiao Fan, Yundai Chen, Feng Cao

**Affiliations:** ^1^Department of Cardiology, Xijing Hospital, Fourth Military Medical University, Xi'an 710032, China; ^2^Department of Cardiology, Chinese PLA General Hospital, Beijing 100853, China

## Abstract

Mesenchymal stem cells (MSCs) have been proposed as a promising cell population for cell therapy and regenerative medicine applications. However, the low retention and poor survival of engrafted cells hampered the therapeutic efficacy of engrafted MSCs. Ghrelin is a 28-amino-acid peptide hormone and is proved to exert a protective effect on the cardiovascular system. This study is designed to investigate the protective effects of ghrelin on engrafted adipose-derived mesenchymal stem cells (ADMSCs) and its beneficial effects with cellular therapy in mice myocardial infarction (MI). Results showed that intramyocardial injection of ADMSCs combining with ghrelin administration inhibited host cardiomyocyte apoptosis, reduced fibrosis, and improved cardiac function. To reveal possible mechanisms, ADMSCs were subjected to hypoxia/serum deprivation (H/SD) injury to simulate ischemic conditions *in vivo*. Ghrelin (10^−8^ M, 33712 pg/ml) improved ADMSCs survival under H/SD condition. Western blot assay revealed that ghrelin increased AKT phosphorylation both *in vivo* and *in vitro*, decreased the proapoptotic protein Bax, and increased the antiapoptotic protein Bcl-2 *in vitro*, while these effects were abolished by PI3K inhibitor LY294002. These revealed that ghrelin may serve as a promising candidate for hormone-driven approaches to improve the efficacy of mesenchymal stem cell-based therapy for cardiac ischemic disease *via* PI3K/AKT pathway.

## 1. Introduction

Ischemic heart disease (IHD) is the leading cause of cardiovascular morbidity and mortality worldwide. In past decades, stem cell therapy provides a promising therapy for tissue regeneration and functional recovery for IHD [1]. However, as was revealed by recent studies, one major challenge for stem cell therapy is the limited survival of engrafted stem cells and its residence in ischemic tissues [2, 3]. This limitation is usually associated with the cells' unconvincing therapeutic efficacy [4]. Accordingly, the need to improve the survival and function of transplanted stem cells should be further stressed.

Ghrelin is a 28-amino-acid peptide hormone which exerts independent cardiovascular protective actions, such as promoting angiogenesis, reducing myocardial ischemic reperfusion injury, enhancing vasodilation, and alleviating heart failure [5–7]. In particular, ghrelin could inhibit inflammatory response and apoptosis in endothelial cells [8]. Our previous study also revealed that ghrelin promoted the proliferation, migration, and nitric oxide (NO) secretion of cardiac microvascular endothelial cells (CMECs) [9].

However, the effects of ghrelin on ADMSC engraftment in ischemic microenvironment have remained unclear. In present study, we imitated hypoxic and ischemic injury with hypoxia/serum deprivation (H/SD) cell model* in vitro* and also utilized a mouse myocardial infarction (MI) model* in vivo* to investigate the effects of ghrelin on ADMSCs in an ischemic setting. Moreover, by establishing ADMSCs which stably expressed molecular imaging reporter genes—firefly luciferase (Fluc) and green fluorescence protein (GFP), we monitored ghrelin's effect on the viability of engrafted ADMSC and* in vivo* possible mechanisms of ghrelin in promoting ADMSC survival.

## 2. Materials and Methods

### 2.1. Animals

Fluc^+^-eGFP^+^ transgenic mice (Tg [Fluc- egfp]) were bred on a FVB/N background, which could constitutively express firefly luciferase (Fluc) and enhanced green fluorescence protein (eGFP) in all tissues and organs, and were used for ADMSCs isolation. Syngeneic female FVB mice with the same genetic background as Fluc^+^-eGFP^+^ transgenic mice (8 weeks old, 20 to 25 g) underwent LAD ligation for the MI model and served as hosts for cellular therapy. All procedures were performed in accordance with the National Institutes of Health Guidelines on the Use of Laboratory Animal. Experimental protocols and animal care methods were approved by the Fourth Military Medical University Committee on Animal Care.

### 2.2. MI Model and Stem Cell Injection

FVB mice (*n* = 80) were divided into 4 groups: (1) sham group (*n* = 20); (2) MI group (MI, *n* = 20); (3) MI + ADMSCs-vector group (ADMSC, *n* = 20); (4) MI + ADMSCs + ghrelin group (ADMSC-ghrelin, *n* = 20). MI was accomplished by ligation of the left anterior descending (LAD) artery with 6-0 silk sutures after left thoracotomy as described before [10]. Ventricle blanching indicated successful occlusion of the vessel. Sham-operated animals served as surgical controls and were subjected to the same procedures as the experimental animals with the exception that the LAD was not ligated. Mortality rates during and after surgery were less than 5% in all groups. 30 minutes later, cell suspensions were directly injected into the ischemic border zone of the myocardium at four different sites (5 *μ*L to each site) with a total volume of 20 *μ*L containing 7 × 10^5^ cells using a Hamilton syringe with a 29-gauge needle. ADMSCs in ADMSC-ghrelin group were pretreated with ghrelin (10^−8^, Phoenix Pharmaceuticals). All surgical procedures were performed blindly by an expert with several years of experience on myocardial model.

### 2.3. Isolation, Cultivation, and Identification of ADMSCs

ADMSCs were isolated with modified procedures described previously [2]. Briefly, adipose tissue was aseptically harvested from Fluc^+^-eGFP^+^ transgenic mice and digested by 0.02% collagenase type I solution for 1 h at 37°C. Then cell suspensions were centrifuged at 200 g for 10 min to separate the stromal cell fraction from adipocytes, the cell pellet was resuspended in DMEM supplemented with 15% fetal bovine serum (FBS). Fresh culture media were changed every 3 days. When MSCs reached 80% of confluence, they were passaged and replated at a concentration of 5 × 10^4^/cm^2^ in cell culture flasks. Cells between third and fifth passage were utilized for further experiments. Cultured ADMSCs were identified by flow cytometry as previously described with minor modifications [2].

### 2.4. Reporter Gene Imaging of ADMSCs^Fluc^+^-eGFP^+^^

Bioluminescence imaging of firefly luciferase reporter gene (Fluc) was performed to determine the correlation between ADMSCs number and Fluc activity* in vitro*. Briefly, ADMSCs of different quantities ranging from 0.1 × 10^5^ to 10 × 10^5^ were seeded into 96-well plates for 3 wells each group, suspended in 500 *μ*L phosphate-buffered saline (PBS), and incubated with reporter probe D-luciferin (2.25 ng/*μ*L, Invitrogen), followed by imaging with Xenogen Kinetic* In vivo* Imaging System (IVIS, Caliper Life Sciences. CA, USA). For* in vitro* cell viability, ADMSCs were plated in 12-well plates. 24 h later, ADMSCs were administered ghrelin (10^−9^, 10^−8^, and 10^−7^ mol/L, resp., equal to 3371.2, 33712, and 337120 pg/mL) or ghrelin with PI3K inhibitor LY294002 (30 *μ*M). After 6 h of either H/SD or normal conditions, cell media were removed from all wells. Cells were incubated with D-luciferin reporter probe (2.25 ng/*μ*L, Invitrogen) and then measured using the IVIS Xenogen Kinetic System (Caliper Life Sciences, USA), using the following imaging parameters: binning at 4, *F*/stop at 1; exposure time with 1 min. For* in vivo* cell viability, engrafted cell was detected using Xenogen* In vivo* Imaging System (IVIS, Caliper Life Sciences, USA) as described previously [11]. Briefly, recipient mice were injected with D-luciferin (150 mg/kg body weight, Caliper, MA, USA) intraperitoneally. Ten minutes later, mice were anesthetized with 2% isoflurane and placed in the imaging chamber. Mice were imaged for 10 min with 1 min acquisition intervals on days 1, 7, 14, and 21 after cell injections. Bioluminescent data were analyzed using Living Image 4.0 software (Caliper, MA, USA) and were quantified as average radiance in photons/s/cm^2^/sr.

### 2.5. ADMSCs Hypoxia/Serum Deprivation Injury

Primary ADMSCs were isolated and cultured as described above. ADMSCs were divided into four groups as follows: control group, H/SD group (H/SD), H/SD + ghrelin (10^−8^ M, 33712 pg/mL) group (Ghrelin), and H/SD + ghrelin (10^−8^ M) + PI3K inhibitor LY294002 (30 *μ*M) group (ghrelin/LY). ADMSCs of the group of ghrelin and ghrelin/LY groups were pretreated with ghrelin (10^−8^ M) before H/SD injury. ADMSCs were stimulated with hypoxia/serum deprivation injury as described previously. Briefly, ADMSCs were exposed to hypoxia (94% N_2_, 5% CO_2_, and 1% O_2_) in an anaerobic system (Thermo Forma) at 37°C for 6 h. In the control group, ADMSCs were maintained at normoxia (95% air, 5% CO_2_) for equivalent periods.

### 2.6. Echocardiography

Transthoracic echocardiography (VEVO 2100, VisualSonic, USA) was performed at 24 hours, 1 week, and 4 weeks after infarction in each group. Left ventricular ejection fraction (LVEF) and fractional shortening (FS) were measured as previously described [12]. All measurements (VEVO 2100, VisualSonic, USA) were averaged for three consecutive cardiac cycles and performed by a blinded investigator.

### 2.7. Histological Assessment of Myocardial Infarction Size

28 days after cell transplantation, myocardial fibrosis was examined by Masson's trichrome staining to indicate infarction area within the left ventricle (LV). Briefly, mice were euthanized and hearts were harvested for histological staining at 5 weeks after cell transplantation. Hearts were prepared in 4% paraformaldehyde and embedded in paraffin before staining. Then heart sections (5 *μ*m) were stained with Masson's trichrome (Sigma-Aldrich; St. Louis, MO). Fibrosis was determined using computer morphometry (Bioquant 98) the collagen area was calculated as a percentage of the total left ventricular myocardial area.

### 2.8. Western Blot Analysis and ELISA Assay

Both cells and myocardium tissues were harvested for Western blot following standard protocol as described previously [2]. Proteins were collected and concentrations were determined using the BCA Protein Assay Kit (Thermo Scientific). Proteins (30 *μ*g/lane) were loaded onto 10% SDS-PAGE gels. After electrophoresis, proteins were transferred to a PVDF Western Blotting membrane (Roche, USA). Membranes were blocked with 5% nonfat dried milk (in TBST) for 2 h at room temperature and then incubated with primary antibody overnight at 4°C (dilution at 1 : 2000 for anti-AKT, 1 : 1000 for phospho-AKT, 1 : 1000 for anti-b-actin, 1 : 200 for anti-Bax, and anti-Bcl-2, all from Cell Signaling Technology, Danvers, MA, USA). After washing and further incubation with appropriate secondary antibody conjugated with horseradish peroxidase for 1 h at room temperature (Cell Signaling Technology), Band intensities were visualized using an enhanced chemiluminescence system (ECL; Amersham). Densitometric analysis of Western blots was carried out using ImageJ software (NIH, Bethesda, MD, USA).

The concentrations of VEGF secreted by ADMSCs were determined by enzyme-linked immunosorbent assay (ELISA) according to the manufacturer's instructions (Sen-Xiong Company, Shanghai, China). In accordance with the manufacturer's instructions, all supernatant was collected, stored at −80°C before measurement and both standards and samples were run in triplicate. OD450 was calculated by subtracting background and standard curves were plotted.

### 2.9. MTT Assay for Cell Viability

The cell viability of ADMSCs was assessed by 3-(4,5-dimethylthiazol-2-yl)-2,5-diphenyltetrazolium bromide (MTT) assay as described [13]. Briefly, ADMSCs were plated in 96-well plates at 1 × 10^4^/well. After H/SD treatment, cells from each group were harvested and incubated with 10 *μ*L MTT (5 g/L) for 4 h. After that, the incubation medium was removed and formazan crystals were dissolved in 150 *μ*L dimethyl sulphoxide (DMSO). The absorbance was determined at a wavelength of 490 nm.

### 2.10. Assessment of Apoptosis

TUNEL staining was performed on ADMSCs as well as myocardial sections (frozen sections) according to the manufacturer's instructions (MEBSTAIN Apoptosis kit II; Takara). A cell death detection kit (Roche) was used to detect apoptotic cells. For detection of total nuclei, the slides were covered with the mounting medium containing DAPI (49,6-diamidino-2-phenylindole) (Sigama, USA). Digital photographs were taken at high magnification using a fluorescent microscopy (Olympus). Cells in which the nucleus was stained were defined as TUNEL positive. The percentage of apoptotic cells was termed the apoptotic index. Caspase-3 activity was measured using a caspase-3 assay kit (Clontech, Mountain View, CA) according to the manufacturer's instructions.

### 2.11. Immunohistochemical Staining for CD31

The density of arteriole was examined in the sections by immunohistochemically staining with anti-CD31 antibody (Sigma, USA), incubated with peroxidase-conjugated streptavidin, stained with DAB, and imaged with microscope (Nikon, Tokyo, Japan). Three high magnification fields within the infarcted region of each section were chosen randomly. Arteriole densities were calculated accordingly. Microvessels in each section were confirmed using the following criteria: (a) being positive for vessel endothelium labeling within the infarct scar; (b) having a visible lumen; and (c) having a diameter between 10 and 100 mm. The density of arteriole was expressed as the quantity of arteriole per mm^2^. The immunoreactive areas for CD31 were analyzed with Image J software.

### 2.12. Statistical Analysis

Results are expressed as mean ± standard deviation (SD). SPSS15.0 (SPSS Inc., USA) was used to perform the one-way analysis of variance (ANOVA) for evaluating the differences among different groups and time points within each group. Pairwise multiple comparisons were to identify the parameters differences between the two groups using Tukey test. Data expressed as proportion was assessed with Chi-square testing. A two-tailed *P* value <0.05 was considered significant. Polynomial regression analysis was performed to evaluate the correlation between cell number and optical radiance* in vitro*.

## 3. Results

### 3.1. Morphology and BLI of ADMSCs^Fluc^+^-eGFP^+^^

**Figure 1:**
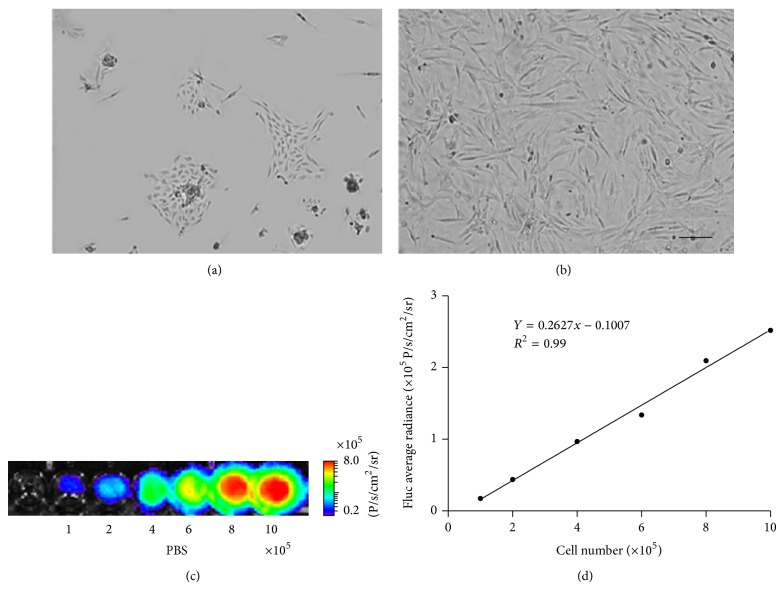
Morphology and bioluminescence imaging (BLI) of ADMSCs^Fluc^+^-eGFP^+^^ *in vitro*. (a) The morphology of ADMSCs after culture for 24 hours; (b) the morphology of ADMSCs after culture for five days (scale bar, 100 *μ*m); (c) bioluminescence imaging of ADMSCs with different cell numbers. Colored scale bars represent optical radiance intensity in photons/second/cm^2^/steradian (P/s/cm^2^/sr); (d) linear correlation of cell quantities with BLI signal was showed.

24 hours after cell isolation, most cells presented a spheroid appearance under (Figure 1(a)). On the sixth day, cells assumed a typical confluent cobblestone morphological appearance (Figure 1(b)). Noninvasive BLI longitudinally revealed the stable expression of firefly luciferase (Fluc) of ADMSCs. Moreover, cells expressed Fluc reporter genes in a number-dependent trend as confirmed by BLI BLI signal intensity of 1.0 × 10^5^ to 1.0 × 10^6^ ADMSCs increase gradually from 1.74 × 104 photons/s/cm^2^/sr to 2.52 × 10^5^ (P/s/cm^2^/sr) (Figure 1(c)). In addition, correlation analysis showed a linear correlation between cell quantities and Fluc signal (correlation coefficient *R* ^2^ = 0.99; linear regression equation: *y* = 0.2627*x* − 0.1007) (Figure 1(d)). These data indicated that BLI of Fluc was a reliable tool to monitor viable transplanted ADMSC quantitatively* in vivo*.

### 3.2. Ghrelin Promoted Viability and Proliferation of ADMSCs under H/SD Injury

**Figure 2:**
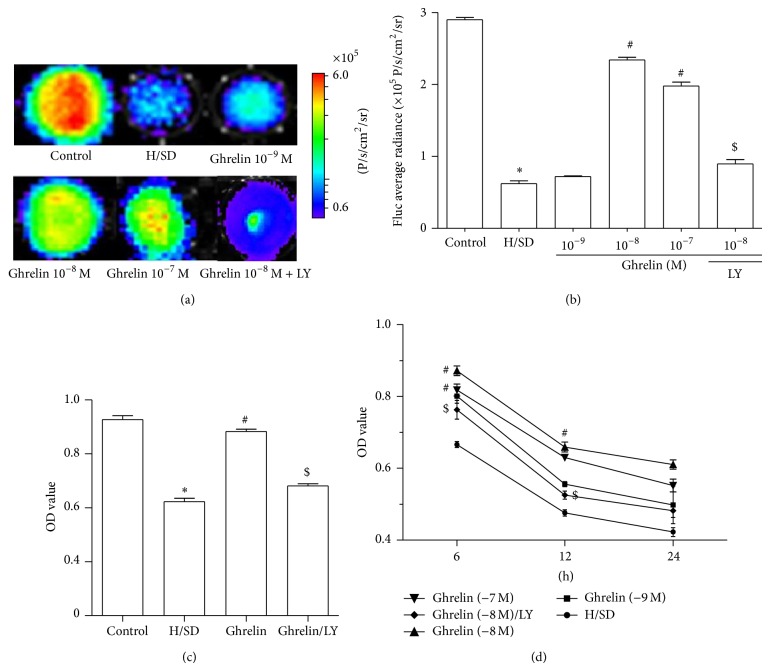
Ghrelin reduced ADMSCs apoptosis and promoted ADMSCs proliferation after H/SD injury. ((a), (b))* In vitro* BLI also confirmed that ghrelin at 10^−8^ mol/L enhanced the impaired viability of ADMSCs after H/SD injury. However, the protective effect of ghrelin was abolished by the PI3K inhibitor LY294002. (c) MTT assay for four groups. (d) MTT assay for H/SD for 6, 12, and 24 hours in different ghrelin pretreatment groups. ^*^ *P* < 0.05 versus control group, ^#^ *P* < 0.05 versus H/SD group, and ^$^ *P* < 0.05 versus ghrelin (10^−8^ M) group.

BLI longitudinally revealed the viability of ADMSCs^Fluc^+^-eGFP^+^^ under H/SD injury. H/SD injury significantly decreased ADMSCs viability after H/SD injury for 6 hours (2.86 × 10^5^ ± 1.73 × 10^4^ versus 6.07 × 10^4^ ± 6.45 × 10^3^, P/s/cm^2^/sr) (*P* < 0.05) and this decrease was significantly reversed (Figure 2(a)) by ghrelin pretreatment at the density of 10^−8^ M (2.30 × 10^5^ ± 6.95 × 10^3^ versus 6.07 × 10^4^ ± 6.45 × 10^3^ P/s/cm^2^/sr, *P* < 0.05), 10^−7^ M (1.96 × 10^5^ ± 1.02 × 10^4^ versus 6.07 × 10^4^ ± 6.45 × 10^3^ P/s/cm^2^/sr, *P* < 0.05), while ghrelin pretreatment at the density of 10^−9^ M showed no statistically significant differences, indicating that ghrelin pretreatment at the concentration of 10^−8^ M and 10^−7^ M could increase the viability of ADMSCs under H/SD injury. The protective effect of ghrelin at 10^−8^ mol/L on the viability of ADMSCs after H/SD injury was abolished by addition of the PI3K inhibitor LY294002 (30 *μ*M, Sigma, USA) (Figures 2(a) and 2(b)).

Cell proliferation was assessed by MTT assay. Different concentrations of ghrelin exerted various effects on ADMSCs proliferation capacity in the condition of normoxia, H/SD for 6 hours, H/SD for 12 hours, and H/SD for 24 hours as assessed by MTT assay (Figures 2(c) and 2(d)). Ghrelin significantly enhanced cell proliferation at concentrations of 10^−8^ M in H/SD 6 hours (0.87 ± 0.02 versus 0.67 ± 0.02, *P* < 0.05), H/SD 12 hours (0.66 ± 0.01 versus 0.47 ± 0.03, *P* < 0.05), and H/SD 24 hours group (0.64 ± 0.02 versus 0.42 ± 0.02, *P* < 0.05) compared with respective H/SD groups. This enhanced proliferation by ghrelin at 10^−8^ mol/L on ADMSCs under H/SD injury was abolished by addition of the PI3K inhibitor LY294002.

### 3.3. Ghrelin Inhibited Apoptosis of ADMSCs

**Figure 3:**
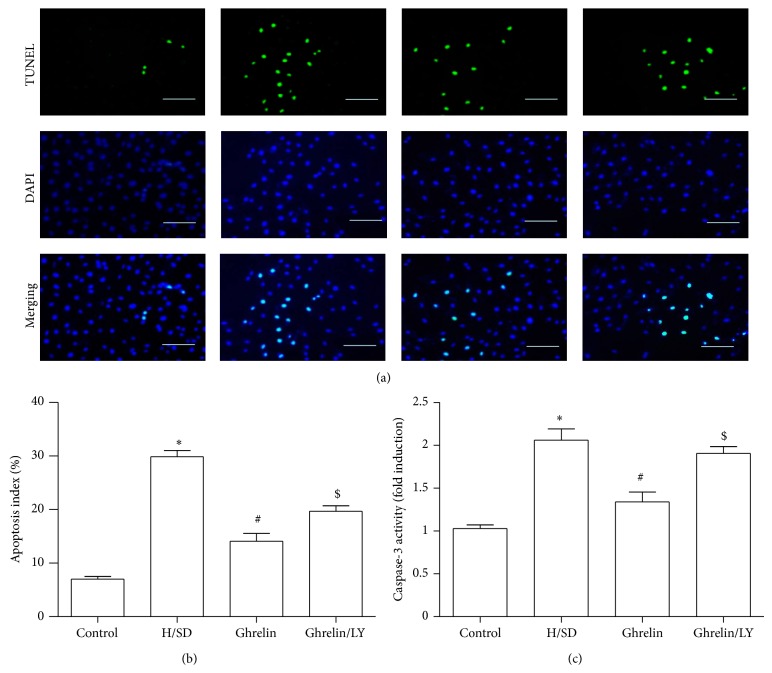
Ghrelin pretreatment reduced ADMSCs apoptosis after H/SD injury. (a) Representative images of TUNEL for apoptotic cells (green, TUNEL; blue, DAPI; scale bar, 100 *μ*m); (b) quantification of TUNEL assay; (c) caspase-3 activity assay confirmed the reduction of ADMSCs apoptosis. ^*^ *P* < 0.05 versus control group, ^#^ *P* < 0.05 versus H/SD group, and ^$^ *P* < 0.05 versus ghrelin (10^−8^ M) group.

TUNEL assay was used to verify whether H/SD induced ADMSCs apoptosis could be reversed by ghrelin. The percentage of apoptotic ADMSCs in the H/SD group significantly increased compared with the control group (29.89 ± 1.98% versus 7.02 + 0.88%, *P* < 0.05). In contrast, pretreatment with ghrelin (10^−8^ M) decreased the apoptotic rates of ADMSCs (14.07 ± 2.57% versus 29.89 ± 1.98%, *P* < 0.05) compared with the H/SD group. However, coincubation with LY294002 abrogated the antiapoptotic effect of ghrelin on ADMSCs (Figures 3(a) and 3(b)). Concurrently, caspase-3 activity assay confirmed the result of TUNEL assay (Figure 3(c)). These data suggest that ghrelin may prevent H/SD injury-induced apoptosis of ADMSCs* via* the PI3K/AKT signaling pathways.

### 3.4. Ghrelin Pretreated ADMSCs Significantly Reduced Fibrosis and Apoptosis after MI

**Figure 4:**
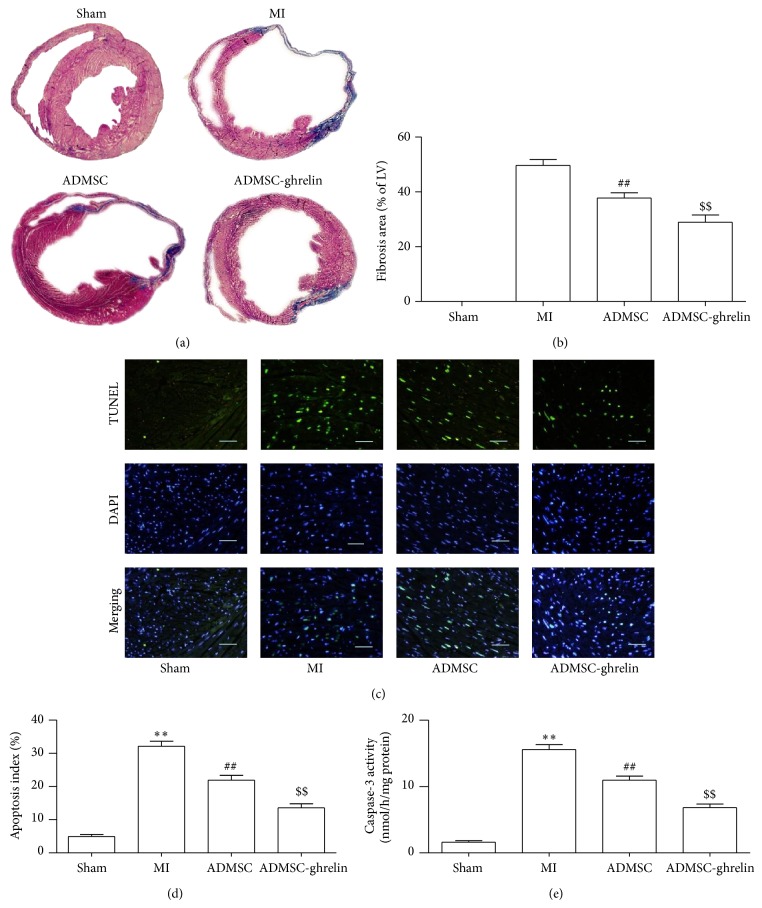
Significant reduction of fibrosis and cardiomyocytes apoptosis after ADMSCs implantation. (a) Representative images of Masson's trichrome staining of each group 4 weeks after MI; (b) quantitative analysis of Masson's trichrome staining; (c) representative images of myocardial sections TUNEL (green, TUNEL; blue, DAPI; scale bar, 100 *μ*m); (d) quantification of apoptotic cells; (e) analysis of caspase-3 activity. ^**^ *P* < 0.05 versus MI group, ^##^ *P* < 0.05 versus MI group, and ^$$^ *P* < 0.05 versus ADMSC group.

Masson trichrome staining was performed to testify if ghrelin combined ADMSCs influenced fibrosis in infarcted myocardium on day 28 after MI (Figures 4(a) and 4(b)). Masson trichrome staining results showed that severe fibrosis was observed in the post-MI hearts of mice without treatment or treated with ADMSCs. Conversely, fibrosis was markedly alleviated in ADMSC-ghrelin group (23.7 ± 3.2%) compared with MI (9.68 ± 4.69%) and ADMSC group (37.79 ± 4.20%) (*P* < 0.05).

TUNEL assay was used to assess the level of apoptosis of cardiomyocytes in infarcted mouse heart (Figures 4(c)-4(d)). As is shown in representative TUNEL images, a significantly higher apoptosis index (AI) was observed in MI group compared with control group (32.12 ± 3.39% versus 4.91 ± 1.43%, *P* < 0.05). A sharp decrease of AI was noted in ADMSC and ADMSC-ghrelin group compared with MI group (21.89 ± 3.27%, 13.57 ± 2.75% versus 32.12 ± 3.39%, *P* < 0.05), indicating that ghrelin pretreated ADMSCs implantation could suppress MI induced apoptosis. Furthermore, this antiapoptotic effect was more pronounced in ADMSC-ghrelin group compared with ADMSC groups (13.57 ± 2.75% versus 21.89 ± 3.27%, *P* < 0.05). Caspase-3 activity assays confirmed that activation of caspase-3 was attenuated in ADMSC-ghrelin group compared with MI and ADMSC group (*P* < 0.05) (Figure 4(e)).

### 3.5. Ghrelin Promoted the Viability of Implanted ADMSCs

**Figure 5:**
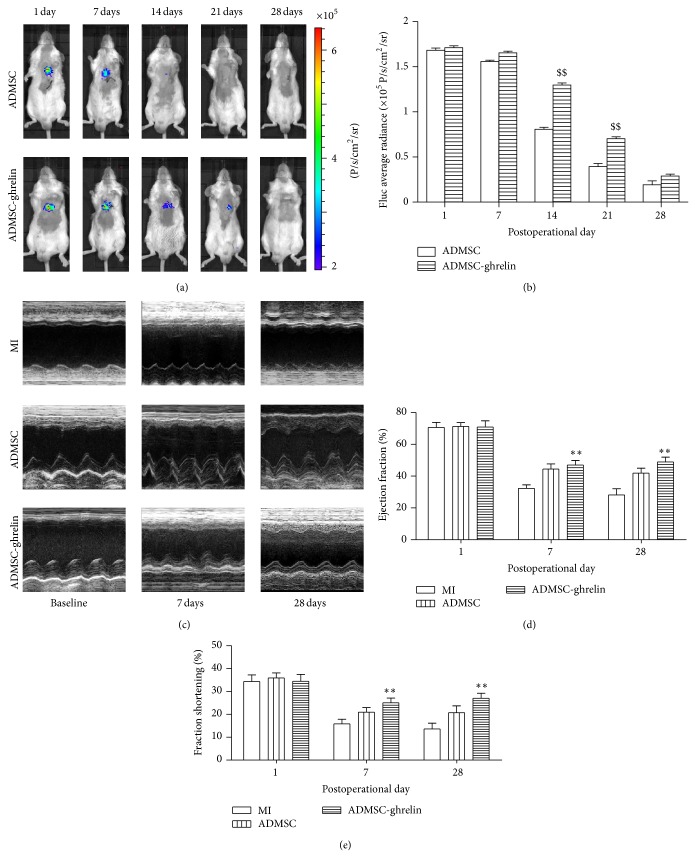
Viability of transplanted ADMSCs and cardiac function after MI. (a)* In vivo* BLI of ADMSCs viability after transplantation; (b) quantitative analysis of BLI. (c) Representative images of M-mode echocardiography. ((d), (e)) Quantitative analysis of cardiac function of ejection fraction (*E*) and fraction shortening (*F*) at baseline and 1 week and 4 weeks after MI. *n* = 10/group, ^**^ *P* < 0.05 versus MI group.

Longitudinal bioluminescence imaging (BLI) was performed to determine the effect of ghrelin on the viability of ADMSCs transplanted into infarcted hearts. After ADMSCs implantation, BLI signals from both groups decreased gradually to background levels after day 21. At postoperative days (POD) 14 and 21, the BLI signals in ADMSC-ghrelin group were 1.30 ± 0.02 × 10^5^ and 0.70 ± 0.02 × 10^5^ photons/s/cm^2^/sr, respectively, significantly higher than that in ADMSC group (0.81 ± 0.02 × 10^5^, 0.40 ± 0.03 × 10^5^ photons/s/cm^2^/sr, *P* < 0.05; Figures 5(a) and 5(b)).

### 3.6. Ghrelin Combined ADMSCs Significantly Improved Cardiac Function after MI

Serial echocardiographic analysis indicated that there was no significant difference in left ventricular ejection fraction (LVEF) and fraction shortening (FS) between all groups at baseline (*P* > 0.05). On POD 7, LVEF decreased significantly in all groups. However, combined therapy of ADMSCs and ghrelin improved cardiac function significantly more than expected. Specifically, by POD 7 and 28 LVEF was improved in the combined therapy group compared to MI groups (POD 7: 46.95 ± 2.92 versus 32.32 ± 2.16% and POD28: 48.924 ± 3.02% versus 28.15 ± 3.92%, Figures 5(c) and 5(d), *P* < 0.05). Similarly, fractional shortening (FS) was significantly improved in the combined therapy group on POD 7 and 28 in contrast to MI groups (POD7: 25.08 ± 2.08 versus 15.84 ± 2.0% and POD28: 27.02 ± 2.20% versus 13.61 ± 2.56%, Figures 5(c) and 5(e), *P* < 0.05).

### 3.7. Ghrelin Regulated AKT Phosphorylation in ADMSCs after H/SD Injury

**Figure 6:**
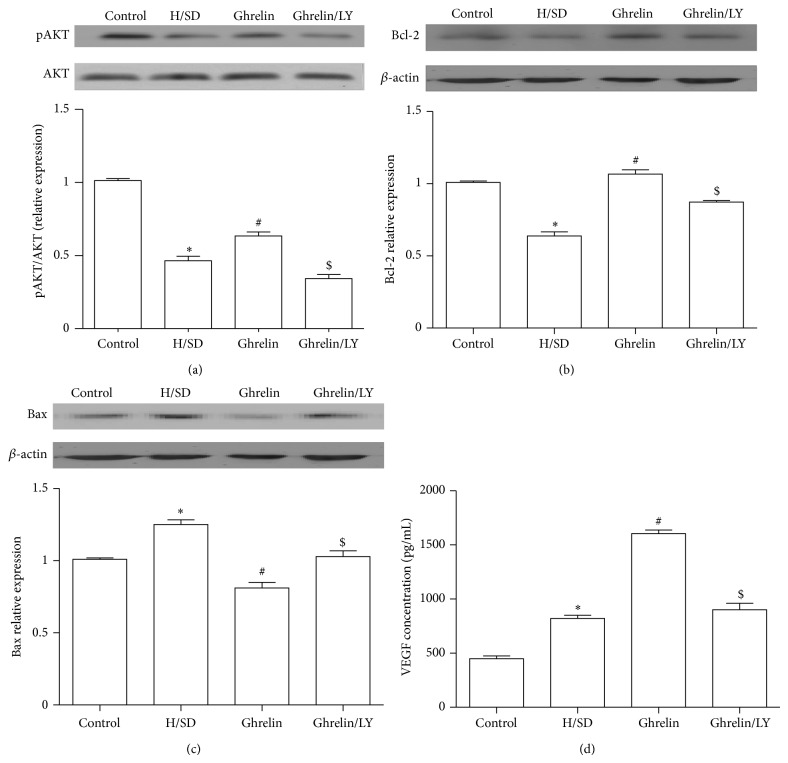
Ghrelin regulated AKT phosphorylation and apoptotic signaling pathways. ((a)–(c)) Western blot and quantification of phosphorylation of AKT (normalized to total AKT), Bcl-2, and Bax respectively (all normalized to control group). (d) Ghrelin increased VEGF secretion in ADMSCs after H/SD injury; ^*^ *P* < 0.05 versus control group, ^#^ *P* < 0.05 versus H/SD group, and ^$^ *P* < 0.05 versus ghrelin (10^−8^ M) group.

Western blot assay (Figure 6(a)) showed that ghrelin administration increased PI3K/AKT phosphorylation in ADMSCs after H/SD injury (*P* < 0.05). The effect of promoting AKT phosphorylation by ghrelin on ADMSCs could be attenuated by LY294002 administration.

### 3.8. Ghrelin Regulated Apoptotic Signaling Pathways

We also analyzed apoptosis associated factors Bcl-2 and Bax protein expression by Western blot assay to figure out whether ghrelin regulated apoptotic signaling pathways (Figures 6(b) and 6(c)). Based on our data, the expression of proapoptotic factor Bax was increased and antiapoptotic factor Bcl-2 was decreased after H/SD injury (*P* < 0.05), while ghrelin inhibited these changes (*P* < 0.05). However, the antiapoptotic effect of ghrelin was eliminated when LY294002 was used (*P* < 0.05), indicating that the antiapoptotic effect of ghrelin was* via* PI3K/AKT pathways.

### 3.9. VEGF Secretion Was Increased by Ghrelin Administration

ELISA assays were performed to evaluate the effect of ghrelin on VEGF secretion in ADMSCs (Figure 6(d)). Data showed that H/SD injury increased VEGF secretion in comparison with the control group (820.90 ± 74.7 versus 449.10 ± 62.50 pg/mL, *P* < 0.05). Furthermore, ghrelin promoted the secretion of VEGF after H/SD, and this effect was abolished by addition of LY294002.

### 3.10. Ghrelin Pretreated ADMSCs Regulated AKT Phosphorylation in Mouse Infarcted Heart

**Figure 7:**
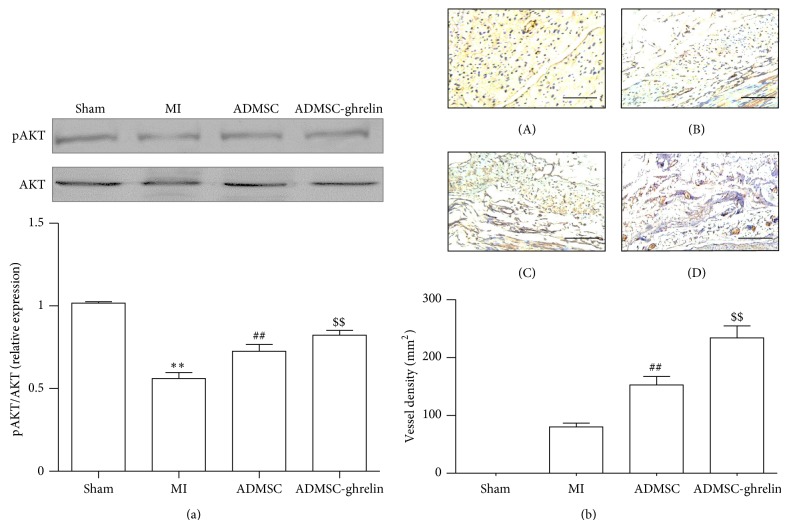
Ghrelin pretreated ADMSCs implantation regulated AKT phosphorylation in mouse infarcted heart and microvessel density in the myocardial infarction sites. (a) ADMSCs implantation regulated AKT phosphorylation in mouse infarcted heart and quantification of phosphorylation of AKT (normalized to total AKT and control group). (b) Microvessel density in the myocardial infarction sites and quantitative analysis. (b) Representative images of myocardial sections from different groups stained with CD31 antibody, (b-A) sham group; (b-B) MI group; (b-C) ADMSC group; and (b-D) ADMSC-ghrelin group. Microvessel densities were statistically compared below between different groups (scale bar = 100 *μ*m). ^**^ *P* < 0.05 versus sham group, ^##^ *P* < 0.05 versus MI group, and ^$$^ *P* < 0.05 versus ADMSC group.

The phosphorylations of AKT in mouse heart of all groups were measured by Western blot assay (Figure 7(a)). Our results showed that ADMSCs implantation increased PI3K/AKT phosphorylation in mouse heart as compared with MI group, and ADMSCs implantation combined ghrelin administration further increased this trend compared with ADMSCs only (*P* < 0.05).

### 3.11. Ghrelin Pretreated ADMSCs Promoted Neovasculature Formation

Arteriole within the infarct was counted to assess the neovascular effect of the different treatments as collateral arterioles are often observed bordering the scar after MI. The results showed that ADMSC (Figure 7(b)-(C), 152.5 ± 25.28/mm^2^) and ADMSC-ghrelin (Figure 7(b)-(D), 233.7 ± 36.23/mm^2^) all resulted in better arteriole density in scar areas than MI group (Figure 7(b)-(B), 79.97 ± 11.18/mm^2^) (*P* < 0.01). The arteriole density of the ADMSC-ghrelin was the highest (*P* < 0.05, resp.).

## 4. Discussion

Mesenchymal stem cells hold promise for cardiovascular regenerative therapy of ischemic heart diseases (IHD) [14]. The success of stem cell-based IHD therapy needs effective cell engraftment and survival rate [15]. However, when stem cells are injected into the infarcted region, most of the cells encounter acute cell death due to the hypoxic and ischemic microenvironment [16]. Ghrelin has been reported to directly exert a protective effect on the cardiovascular system [17, 18]. Our present study has verified for the first time the beneficial effects of ghrelin on adipose tissue-derived stromal cells (ADMSCs) based IHD therapy. Our results revealed that ADMSC-ghrelin significantly reduced cardiac fibrosis, decreased cardiomyocyte apoptosis, and improved cardiac function after MI injury. Moreover, ghrelin increased the survival of transplanted ADMSCs in the regional myocardial tissue. Furthermore, both* in vivo* and* in vitro* results verified that ghrelin exerts the protective effect on ADMSCs and infarcted heart partly through the activation of PI3K/AKT signaling pathways.

Ghrelin is a 28-amino acid peptide secreted by the stomach, which serves as an endogenous ligand for growth GHSR [5]. Numerous investigations have been done recently suggesting that ghrelin is capable of exerting cardioprotective effects. Ghrelin was reported to have anti-inflammatory effects, specifically* via* suppression of chemotactic factors such as IL-8 and MCP-1 that are normally induced by TNF*α*-mediated NF-*κ*B activation [8]. Ghrelin also inhibited the adherence of U937 monocytes to HUVECs (human umbilical vein endothelial cells), another mechanism by which ghrelin may suppress the development of early atherosclerosis [19]. Moreover, ghrelin could also inhibit high glucose-induced (33.3 mM, 72 h) apoptosis of HUVECs, possibly by decreasing the concentration of ROS reactive oxygen species [20]. Protective as ghrelin seemed to be, we were curious to know whether ghrelin could also exert a protective effect on ADMSCs in an ischemic setting. In our study, we found that pretreatment with ghrelin could induce ADMSC proliferation, inhibit apoptosis, and increase VEGF secretion under H/SD injury* in vitro*; moreover, ghrelin could exert a protective effect on mesenchymal stem cells (ADMSCs) in the model of MI in the mouse heart, indicating that ghrelin may be a favorable factor in stem cell-based IHD therapy. Similarly, some previous reports have indicated that ghrelin significantly increased the proliferation of C3H10T1/2 cells at the concentration of 10^−13^ and 10^−11^ M. Ghrelin also exerted an antiapoptotic effect on C3H10T1/2 cells by decreasing caspase-3 activity significantly at concentrations between 10^−13^ and 10^−7^ M [21]. Ghrelin could serve as an autocrine signal regulating skeletal myogenesis, exogenous ghrelin stimulation was shown to regulate myoblast migration and proliferation, and the addition of ghrelin to the differentiation medium increased myogenic differentiation of L6E9 cells [22]. However, other studies have documented that 1 *μ*M ghrelin induced apoptosis in colorectal adenocarcinoma cells by inhibiting the ubiquitin-proteasome system and by activating autophagy, with p53 having an “interactive” role [23]. In addition, ghrelin induced a significant inhibition of cell proliferation in MCF7 cells, at a concentration of 1 × 10^−6^ M [24]. From our standpoint, ghrelin may act as either antiapoptotic or proapoptotic factor and may enhance or inhibit proliferation in different cells, suggesting that these effects are cell type dependent and are presumably affected by specific cell microenvironment.

Additionally, we found the presence of ghrelin increased the secretion of VEGF of ADMSCs under H/SD injury. VEGF was recognized as a central mediator of angiogenesis [25]. We previously reported that VEGF enhanced the functional survival of donor cells in ischemic myocardium suggesting VEGF secretion is a protective response of ADMSCs to ischemia* in vivo* and hypoxic stimuli* in vitro* [26]. VEGF primarily activates the VEGFR2 (KDR/Flk-1) tyrosine kinase, a key regulator of proangiogenic and antiapoptotic responses [27]. Activation of VEGF/VEGFR2 facilitated the functional survival of ADMSCs. This may be one of the possible mechanisms by which ghrelin enhance proliferation of ADMSCs* in vivo*.

Although previous studies demonstrated that the ADMSC together with its secretome could enhance tissue regeneration in ischemic models, the fate of ADMSCs in ischemic settings could not been fully illuminated using traditional cell tracking techniques. Furthermore, the longitudinal therapeutic efficacy of the engrafted cells was uncertain. Previously, we demonstrated that molecular imaging strategy provided valuable insight into the* in vivo* kinetics of engrafted cells [28]. Namely, BLI is an accurate and sensitive approach for noninvasive stem cell tracking of as few as 500 cells. By BLI, we longitudinally and spatiotemporally visualized the viability of ADMSCs both* in vitro* and* in vivo*, which favorably provided an incremental benefit in monitoring the effects of ghrelin on ADMSCs.

However, there are still some limitations in our present study. Since ghrelin peptides circulate in two distinct forms, AG (acylated ghrelin) and UAG (unacylated ghrelin), they may play different roles in the pathogenesis of specific diseases [29]. In our study we chose mouse UAG (the most abundant form of ghrelin in plasma, amino acids sequence: GSSFLSPEHQKAQQRKESKKPPAKLQRP) as our interesting target according to the previous study [30]. However, we did not compare these two forms of ghrelin in their effects of cellular therapy, which is one of our study limitations. To elucidate systematically this issue, future studies will compare the roles of AG and UAG in ADMSC based mice MI therapy.

## 5. Conclusions

This study demonstrated that ghrelin pretreatment promoted the proliferation and inhibited apoptosis of ADMSCs under H/SD injury, improving therapeutic efficacy of ADMSC based stem cell therapy for IHD. It is suggested that ghrelin may potentially serve as a potent agent for a hormone-driven strategy to facilitate the progression of stem cell-based transplantation therapy for ischemic disease with clinical perspective.
